# One-Year and Five-Year Outcomes of Transcatheter Aortic Valve Replacement or Surgical Aortic Valve Replacement in a Taiwanese Elderly Population

**DOI:** 10.3390/jcm12103429

**Published:** 2023-05-12

**Authors:** Po-Han Lin, Hao-Ji Wei, Shih-Rong Hsieh, Hung-Wen Tsai, Chu-Leng Yu, Wen-Lieng Lee, Yung-Szu Wu

**Affiliations:** 1 Division of Cardiovascular Surgery, Cardiovascular Center, Taichung Veterans General Hospital, Taichung 407219, Taiwan; 2 Division of Cardiovascular Surgery, Department of Surgery, Chaiyi Branch, Taichung Veterans General Hospital, Chaiyi 60090, Taiwan; 3 Institute of Clinical Medicine, National Yang-Ming Chiao Tung University, Taipei 112304, Taiwan; 4 Cardiovascular Center, Taichung Tzu Chi Hospital, Taichung 427213, Taiwan; 5 Division of Interventional Cardiology, Cardiovascular Center, Taichung Veterans General Hospital, Taichung 407219, Taiwan

**Keywords:** transcatheter aortic valve replacement, surgical aortic valve replacement, body mass index, mortality, acute kidney injury

## Abstract

Background: The aim of our study was to provide real-world data on outcomes for elderly Taiwanese patients who underwent transcatheter aortic valve replacement or surgical aortic valve replacement in different risk groups. Methods: From March 2011 through December 2021, 177 patients with severe aortic stenosis who were ≥70 years old and had undergone TAVI (transcatheter aortic valve implantation) or SAVR (surgical aortic valve replacement) in a single center were divided by STS score (<4%, 4–8% and >8%) into three different groups. Then, we compared their clinical characteristics, operative complications, and all-cause mortality. Results: In all risk groups, there were no significant differences in in-hospital mortality, or 1-year and 5-year mortality between patients in the TAVI and SAVR groups. In all risk groups, patients in the TAVI group had shorter hospital stay and higher rate of paravalvular leakage than the SAVR group. After univariate analysis, BMI (body mass index) < 20 was a risk factor for higher 1-year and 5-year mortality. In the multivariate analysis, acute kidney injury was an independent factor for predicting worse outcomes in terms of 1-year and 5-year mortality. Conclusions: Taiwan elderly patients in all risk groups did not have significant differences in mortality rates between the TAVI and the SAVR group. However, the TAVI group had shorter hospital stay and higher rate of paravalvular leakage in all risk groups.

## 1. Introduction

Transcatheter aortic valve implantation (TAVI) and surgical aortic valve replacement (SAVR) are standard treatments for severe aortic stenosis, especially for patients older than 65 years [1,2]. According to the Society of Thoracic Surgeons (STS) scores, patients are divided into high, intermediate, and low-risk groups. TAVI is the recommended treatment for high-risk patients [1,2,3]. However, for intermediate and low-risk patients, TAVI or SAVR are standard treatments [4,5] because these treatments have different benefits and disadvantages depending on a patient’s individual condition, which is evaluated by a heart team.

Body mass index (BMI) is one of several factors that can affect the results of patients with TAVI or SAVR [6,7]. Previous research on cardiac surgeries have revealed that surgical outcomes vary among patients with different body mass indices (BMIs) [8,9]. Several studies demonstrated that extreme obesity and underweight were significantly associated with early major adverse clinical outcomes [9,10,11]. Moreover, one of the differences between elderly populations in Taiwan and Western countries is the proportion of people with normal and overweight BMI [12,13]. Previous studies revealed that overweight patients have better results than patients with BMI less than 20 [1,2]. The PARTNER trials are three of the largest randomized control trials comparing results between TAVI and SAVR. The average BMI of the patients in these trials falls within the overweight range [3,4,5]. The largest database of Japanese patients with TAVI and SAVR shows a higher proportion of normal BMI in the short-term results [3]. However, in this research, patients were not divided into different risk groups. Therefore, there were no data on the differences in outcomes of patients of an Asia-Pacific population between TAVI and SAVR in high-, intermediate-, and low-risk groups.

In our retrospective study, data from patients with SAVR and TAVI have been collected over the past 10 years. The purpose of this study was to provide real-world data on the mid-term outcomes of elderly Taiwanese patients in different risk groups who underwent TAVI or SAVR.

## 2. Materials and Methods

All data used in the retrospective cohort study were obtained from patients’ medical records in our hospital (paper and electronic files) and telephone surveys. Patients were treated with TAVI or SAVR in a single center between March 2011 and December 2021. Institutional review board approval was obtained for this study.

**Figure 1:**
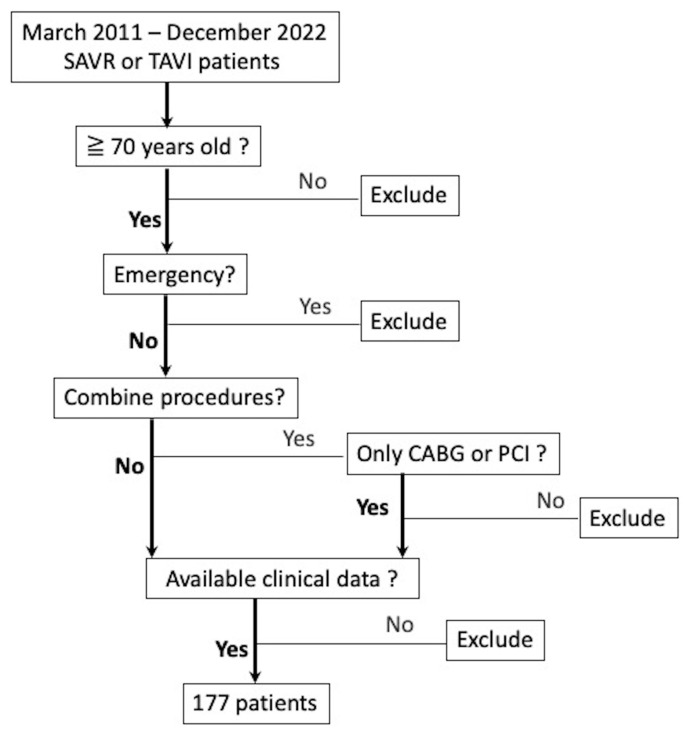
The flowchart of patient selection.

The present study employed specific inclusion and exclusion criteria to recruit eligible participants. Inclusion criteria required the identification of patients aged 70 years or older who received transcatheter aortic valve implantation (TAVI) or surgical aortic valve replacement (SAVR) at our hospital. Among these patients, those who underwent coronary artery bypass grafting (CABG) or percutaneous coronary intervention (PCI) in the same admission were deemed eligible for study enrollment. Conversely, patients who underwent concomitant valve surgery, thoracic aortic surgery, or emergency surgery were excluded from the study, as per the predefined exclusion criteria (Figure 1).

Eligible patients were divided into three main groups, based on STS scores: <4% (low risk), 4–8% (intermediate risk), and >8% (high risk). In each group, they were separated into two subgroups by treatments: TAVI and SAVR. In the TAVI group, we used Medtronic Corevalves and Edward Sapien valves. All TAVI patients were treated using the transfemoral approach. In the SAVR group, we used mechanical valves (On-X prosthetic heart valve, Cryolife, Inc., Kennesaw, GA, USA), bovine valves (Carpentier-Edwards PERIMOUNT Magna Ease aortic heart valve, Edwards Lifescience Corporation, Irvine, CA, USA), and porcine valves (Epic_TM_ aortic valve bioprothesis, St. Jude Medical, Inc., Saint Paul, MN, USA). All SAVR patients received a conventional sternotomy.

The clinical data for each patient included the following: a medical history of the underlying disease, surgical reports, cardiac ultrasound reports, laboratory analyses, and fatal events. The definition of acute kidney injury (AKI) was based on the RIFLE criteria. In this study, AKI is defined as a two-fold increase or more in serum creatinine or a decrease of over 50% in urine output. The primary endpoint was death from any cause.

Statistical analysis was performed using the Social Sciences Statistical Package (IBM SPSS version 22.0; International Business Machines Corp., New York, NY, USA). Continuous variables were reported as average and standard deviation, and categorical data were reported as number and percentage. Data were analyzed using the Kruskal–Wallis test, Mann–Whitney U-test, Chi-Square test, F-test and Fisher’s exact test.

We examined the potential preoperative, operative, and postoperative risk factors for mortality using univariate and multivariate modelling. The factors were selected based on clinical relevance or when the significance of the univariate association exhibited a *p*-value less than 0.5. Event-free survival was calculated using the Kaplan–Meier method. Independent predictors of long-term survival were determined by the Cox proportional hazards model with 95% confidence intervals (CIs). *p*-Values less than 0.05 were considered statistically significant.

## 3. Results

During the study, our hospital performed TAVI on 115 patients and surgical aortic valve replacement on 672 patients. There were 177 patients that met the inclusion and exclusion criteria. The average age was 80.2 ± 6.02 years in all patients, and the average BMI was 24.6 ± 4.05. According to the definition of STS scores, there were 57 patients in the low-risk group, 65 patients in the intermediate-risk group, and 55 patients in the high-risk group. The baseline demographic and clinical data for the patients are presented in Table 1.

In these three different risk groups, the average age of the patients with TAVI was older than that of the SAVR group (low-risk group: 79.6 ± 3.8 vs. 75.4 ± 3.9, *p* < 0.001; intermediate-risk group: 83.1 ± 5.2 vs. 79.5 ± 4.6, *p* = 0.015; high-risk group: 83.9 ± 6.2 vs. 74.3 ± 4.1, *p* < 0.001). All showed no significant differences in STS score and EuroSCORE II between the TAVI and SAVR groups. In addition, in the low-risk and intermediate-risk groups, fewer patients had heart failure symptoms in the TAVI group than in the SAVR group (low-risk group: 50% vs. 91.9%, *p* = 0.001; intermediate risk group: 67.6% vs. 92.9%, *p* = 0.014).

Regarding the treatment of coronary artery disease in these three risk groups, percutaneous coronary intervention (PCI) was used in more patients in the TAVI group than in the SAVR group. In the intermediate-risk group, patients with previous PCI comprised 51.4% of the TAVI group and 25% of the SAVR group (*p* = 0.032). In the high-risk group, 57.8% and 10% of the patients in the TAVI group and the SAVR group had previous PCI (*p* = 0.012). In the low-risk group, 5% and 0% of patients in the TAVI and SAVR groups, respectively, had PCI in the past 12 months (*p* = 0.004). In the high-risk group, 53.3% of patients in the TAVI group and 10% of patients in the SAVR group received PCI in the past 12 months (*p* = 0.015).

The intraoperative and postoperative outcomes are shown in Table 2. In the low- and intermediate-risk groups, fewer patients had CABG in the TAVI group than in the SAVR groups (low-risk: 0% vs. 18.9%, *p* = 0.045; intermediate-risk: 0% vs. 21.4%, *p* = 0.005). In all risk groups, more patients had paravalvular leakage in the TAVI group than in the SAVR groups. All recorded instances of paravalvular leakage were mild paravalvular leakage (low-risk: 40% vs. 0%, *p* < 0.001; intermediate-risk: 27% vs. 0%, *p* = 0.007; high-risk: 42.2% vs. 0%, *p* = 0.010).

In the low- and intermediate-risk groups, more patients in the TAVI groups had extubation in the operating theater than in the SAVR groups (Low-risk: 80% vs. 0%, *p* < 0.001; intermediate-risk: 43.2% vs. 0%, *p* < 0.001). Additionally, in these two risk groups, the TAVI groups had shorter ventilator duration than the SAVR groups (low-risk: 4.3 ± 0.6 vs. 31.7 ± 62.3 h, *p* = 0.001; intermediate-risk: 36.8 ± 126.6 vs. 474.4 ± 1678.3 h, *p* < 0.001). In all risk groups, patients in the TAVI group had shorter hospital stays than those of the SAVR group (low-risk: 7.9 ± 5.9 vs. 10.4 ± 3.8, *p* = 0.005; intermediate-risk: 7.2 ± 7.7 vs. 17.2 ± 17.2, *p* < 0.001; high-risk: 15.0 ± 24.1 vs. 22.5 ± 20.7, *p* = 0.029).

**Figure 2:**
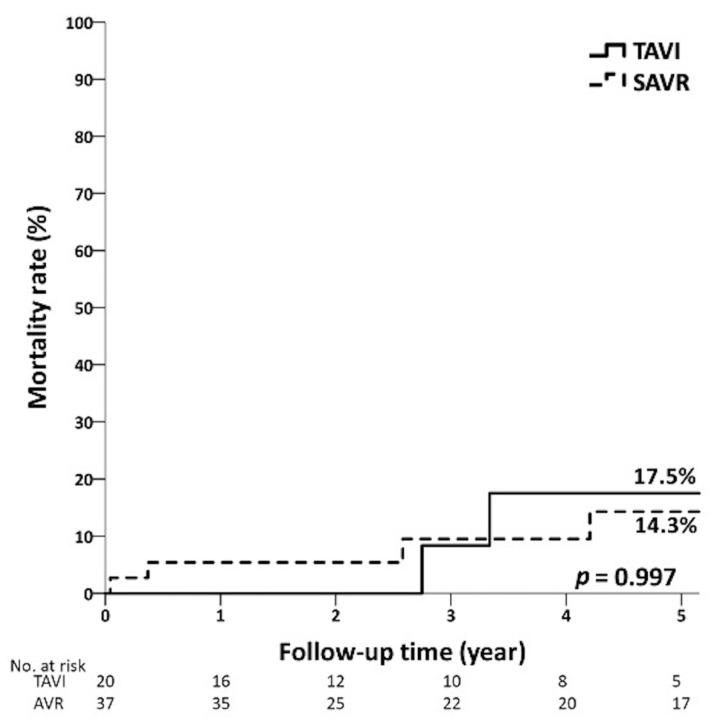
Five-year mortality curves of TAVI and SAVR patients in low-risk group.

**Figure 3:**
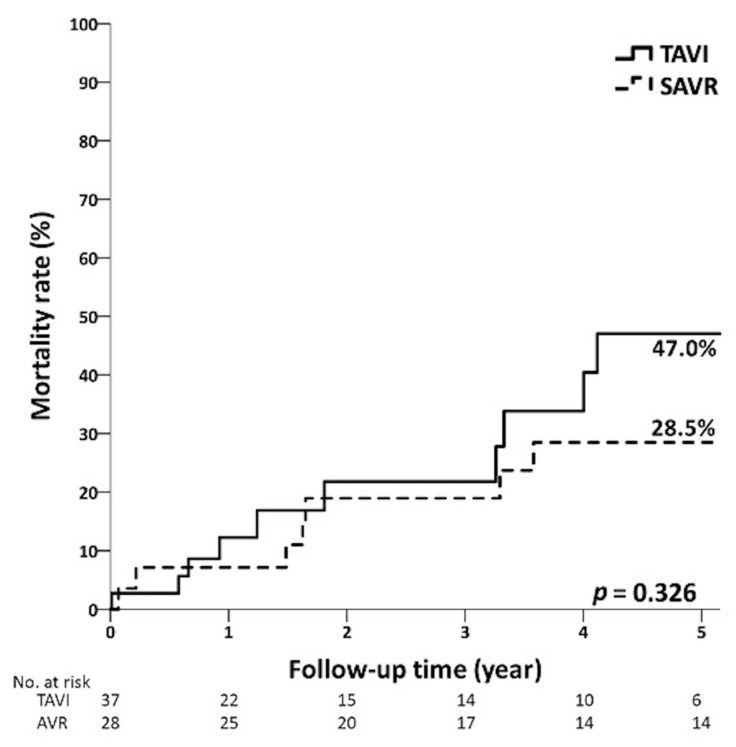
Five-year mortality curves of TAVI and SAVR patients in intermediate-risk group.

**Figure 4:**
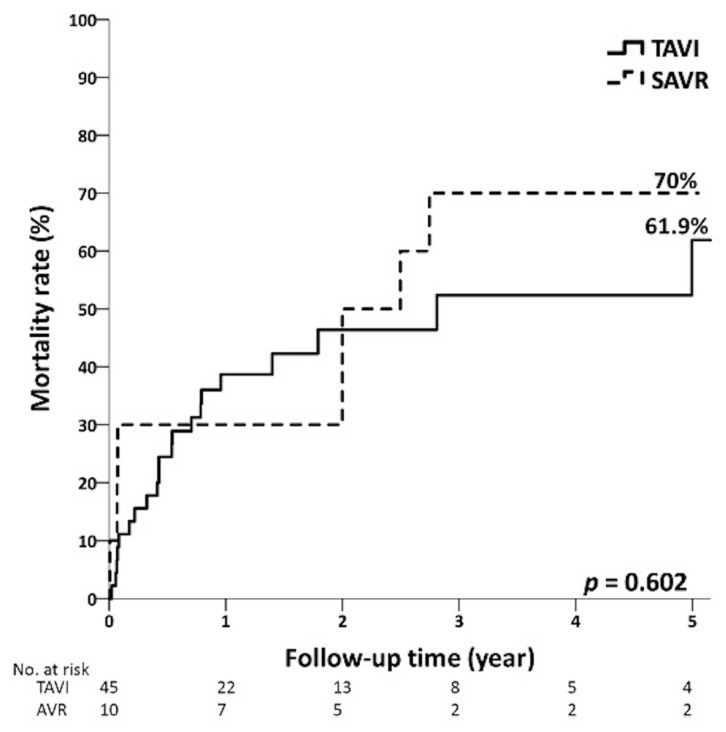
Five-year mortality curves of TAVI and SAVR patients in high-risk group.

The average follow-up times were 3.4 ± 2.3 years and 4.7 ± 3.2 years (*p* = 0.155) in the TAVI group and the SAVR group with low risk, respectively. Figure 2 shows that the 5-year mortality rates of low-risk patients in the TAVI and SAVR groups were 17.5% and 14.3%, respectively (*p* = 0.997). In the intermediate-risk group, the average follow-up time of patients in the TAVI group and SAVR group were 2.6 ± 2.5 and 4.2 ± 2.6 years (*p* = 0.012). There were no significant differences between the TAVI and SAVR groups in the overall mortality, 1-year mortality, and 5-year mortality (Figure 3). In the high-risk group, the average follow-up times of the TAVI and SAVR group were 1.7 ± 1.8 years and 2.2 ± 1.9 years (*p* = 0.458). The overall mortality of the TAVI group (46.7%) was lower than that of the SAVR group (90.0%, *p* = 0.015). No significant differences were observed in 1-year mortality and 5-year mortality (Figure 4) between the TAVI and SAVR groups in the high-risk group.

The results of the Cox regression analysis of univariate and multivariate risk factors for mortality are documented in Table 3. For in-hospital mortality, patients with diabetes mellitus (DM) had a 3.71 times greater risk than non-diabetic patients (HR 3.71 (1.12–12.32), *p* = 0.032) in the multivariate risk factor analysis. Multivariate risk factors that increase the risks of 1-year mortality include STS > 8% (HR 6.03 (1.20–30.46), *p* = 0.030), re-intubation (HR 4.19 (1.48–11.89), *p* = 0.007), and acute kidney injury (HR 4.12 (1.74–9.73), *p* = 0.001). In the multivariate analysis of risk factors for 5-year mortality, STS > 8% (HR 3.13 (1.01–9.74), *p* = 0.048), PAOD (HR 2.96 (1.13–7.77), *p* = 0.028), re-intubation (HR 3.87 (1.53–9.82), *p* = 0.004), and acute kidney injury (HR 3.98 (1.95–8.15), *p* < 0.001) showed statistically significant differences.

## 4. Discussion

In this retrospective study conducted at our center, Taiwanese elderly patients were divided into three risk groups and the long-term clinical results of the TAVI and SAVR groups were compared. In high-risk patients, the TAVI group had lower overall mortality and shorter hospital stay than the SAVR group. In contrast, in intermediate-risk patients, the SAVR group had a lower mortality rate than the TAVI group. Based on our limited data, there appeared to be no significant differences in 1-year mortality between our study and the PARTNER trials for overweight patients in these three risk groups [4,5,6].

### 4.1. Body Mass Index

Moreover, the risk of mortality in our study of elderly Taiwan patients was similar to that of overweight patients in Western countries. However, the results of our univariate regression analysis revealed that patients with BMI < 20 had increased risks of mortality at 1 year and 5 years. Underweight can be caused by multiple factors, which may increase the long-term mortality rate in underweight patients, particularly after SAVR or TAVI [1,14]. A study by Sannino A et al. mentioned that underweight patients also have higher long-term mortality rates after TAVI compared to patients with normal body weight. Additionally, underweight patients are more likely to experience major vascular complications and major or life-threatening bleedings during the procedure, based on VARC-2 definitions. On the other hand, overweight patients have similar procedure complications to those with normal body weight [7]. Thus, BMI < 20 could be a factor for predicting worse outcomes in both the TAVI and SAVR groups.

### 4.2. Diabetes Mellitus

Patients with diabetes mellitus in our study had a 3.71 times greater risk of 30-day mortality than non-diabetes patients after multivariate regression analysis. It remains controversial as to whether DM could increase the risk of poor outcomes after TAVI. In some studies, DM patients after TAVI did not have significantly reduced survival rates in short- and mid-term outcomes [15,16,17]. However, other studies showed that DM was significantly associated with poor outcomes after TAVI. In these studies, insulin-treated DM patients had a higher risk of death than nondiabetic patients [18,19,20,21]. The severity of diabetes after TAVI could be the key factor in the patients’ outcomes.

### 4.3. Peripheral Arterial Obstructive Disease

Peripheral arterial obstructive disease (PAOD) was shown to be a risk factor for 5-year mortality after multivariate analysis in our study. In terms of STS score and EuroScore, PAOD is a risk factor that can increase surgical mortality. For TAVI candidate patients under preoperative survey, PAOD is an important item to evaluate, especially for femoral vascular access. Furthermore, based on this meta-analysis, TAVI patients with PAOD had short- (HR 1.36, 95% confidence interval [CI] 1.13–1.63, *p* = 0.0009), mid- (HR 1.18, 95% CI 1.08–1.30, *p* = 0.0005), and long-term (HR 1.36, 95% CI 1.24–1.48, *p* < 0.0001) outcomes that showed a higher risk of mortality [22]. Therefore, PAOD could also be a risk factor to predict short- and long-term outcomes of patients after TAVI [23,24].

### 4.4. Acute Kidney Injury

Acute kidney injury (AKI) after TAVI and SAVR in our study was an independent risk factor for 1-year and 5-year mortality (HR 4.12 (1.74–9.73), *p* = 0.001; HR 3.98 (1.95–8.15), *p* < 0.001). In a meta-analysis, the data revealed that the incidence of AKI was lower after TAVI than after SAVR (7.1% vs. 12.1%, OR 0.52 (0.39–0.68), *p* < 0.001, I^2^ = 57%) [25]. However, AKI can impede the benefit of TAVI, because patients with AKI after TAVI were more likely to suffer complications, such as hyperkalemia, pulmonary edema, metabolic acidosis, infection, and red blood transfusion for up to 6 months after intervention [26]. Therefore, the short- and long-term outcomes of TAVI patients with AKI are worse than those of the patients without AKI [27,28,29].

### 4.5. Research Contributions

This study is indeed a relatively small research outcome, and although the results are comparable to those of other large studies, it should be noted that there is a lack of relevant data on risk group-based grouping in Asian studies. According to our study, we found that Taiwan’s elderly patients had similar outcomes to those of overweight patients in the American population. Therefore, it is reasonable to postulate that patients in the TAVI and SAVR groups with low BMI had worse outcomes than patients with normal and overweight BMI in both groups [1]. Thus, it is important to carefully evaluate low-BMI patients undergoing TAVI or SAVR to determine whether the benefits outweigh the potential harms. For patients with underlying conditions such as DM or PAOD, a multi-disciplinary team is necessary for careful evaluation and management. Additionally, reducing the incidence of AKI could improve the clinical outcomes in these patients.

Furthermore, under the constraints of the health insurance system, our study cannot provide a large number of research outcomes. However, it is worth noting that because of the government-established health insurance system, we can track the postoperative outcomes of the vast majority of patients and ensure that they receive timely treatment if complications occur after surgery. Thus, our study results suggest that under such a health insurance system, we can provide surgical outcomes similar to those in Western countries in a different medical environment.

### 4.6. Study Limitation

However, there were some limitations in this study. This was a retrospective study with a small number of patients and was conducted in a single institution, and some patients had a mechanical aortic valve prothesis. These factors could affect the long-term outcomes of some patients, such as complications from the use of mechanical valve. Therefore, we intend to collect data from more patients and will divide patients by type of prothesis in future studies.

## 5. Conclusions

Our study, though limited in scope and size, suggest that Taiwan’s elderly patients in all risk groups may not show significant differences in 1-year and 5-year mortality rates between the TAVI group and the SAVR group. In high-risk patients of the TAVI group (46.7%), the overall mortality rate was higher than in patients of the SAVR group (90.0%, *p* = 0.015). In contrast, the TAVI group had a shorter hospital stay and a higher rate of paravalvular leakage than patients in the SAVR groups in all risk groups. Multidisciplinary teams should carefully evaluate and manage patients with a BMI < 20 or a medical history of DM or PAOD who are undergoing TAVI or SAVR.

## Figures and Tables

**Table 1:** Patient baseline characteristics.

	STS Score < 4%					STS Score 4–8%					STS Score > 8%				
Characteristics	TAVI (*n* = 20)		SAVR (*n* = 37)		*p* Value	TAVI (*n* = 37)		SAVR (*n* = 28)		*p* Value	TAVI (*n* = 45)		SAVR (*n* = 10)		*p* Value
Age	79.6	±3.8	75.4	±3.9	<0.001 **	83.1	±5.2	79.5	±4.6	0.015 *	83.9	±6.2	74.3	±4.1	<0.001 **
Gender					0.898					0.861					1.000
Female	11	(55.0%)	21	(56.8%)		23	(62.2%)	18	(64.3%)		26	(57.8%)	6	(60.0%)	
Male	9	(45.0%)	16	(43.2%)		14	(37.8%)	10	(35.7%)		19	(42.2%)	4	(40.0%)	
Body height (cm)	155.4	±7.1	157.0	±8.7	0.525	155.1	±7.2	153.3	±8.0	0.323	153.3	±8.2	153.7	±5.4	0.662
Body weight (kg)	63.1	±9.1	66.3	±12.7	0.682	59.2	±10.5	57.0	±9.2	0.317	53.9	±8.9	53.6	±10.5	0.777
Body mass index	26.1	±2.8	26.9	±4.7	0.907	24.5	±3.5	24.3	±3.7	0.731	22.9	±3.6	22.7	±4.6	0.600
Body mass index < 20 ^f^	1	(5.0%)	1	(2.7%)	1.000	4	(10.8%)	5	(17.9%)	0.483	10	(22.2%)	3	(30.0%)	0.685
STS score (%)	2.8	±0.7	2.5	±0.7	0.201	5.9	±1.3	5.4	±1.2	0.131	15.4	±6.5	14.2	±5.0	0.678
Euroscore II (%)	2.5	±1.1	3.3	±2.4	0.504	5.9	±4.4	6.9	±5.7	0.781	10.4	±8.9	13.4	±8.6	0.150
Symptoms and Signs															
Heart failure ^f^	10	(50.0%)	34	(91.9%)	0.001 **	25	(67.6%)	26	(92.9%)	0.014 *	40	(88.9%)	10	(100%)	0.572
Syncope ^f^	1	(5.0%)	3	(8.1%)	1.000	3	(8.1%)	3	(10.7%)	1.000	5	(11.1%)	3	(30.0%)	0.149
Angina	8	(40.0%)	16	(43.2%)	0.813	11	(29.7%)	15	(53.6%)	0.052	14	(31.1%)	6	(60.0%)	0.144
Mild symptom ^f^	8	(40.0%)	3	(8.1%)	0.011 *	8	(21.6%)	0	(0%)	0.008 **	5	(11.1%)	0	(0%)	0.572
NYHA					<0.001 **					0.001 **					0.072
1	3	(15.0%)	0	(0%)		5	(13.5%)	0	(0%)		1	(2.2%)	0	(0%)	
2	10	(50.0%)	4	(10.8%)		15	(40.5%)	2	(7.1%)		18	(40.0%)	0	(0%)	
3	6	(30.0%)	32	(86.5%)		15	(40.5%)	21	(75.0%)		16	(35.6%)	5	(50.0%)	
4	1	(5.0%)	1	(2.7%)		2	(5.4%)	5	(17.9%)		10	(22.2%)	5	(50.0%)	
Cardiac ultrasonography															
Left ventricular ejection fraction (%)	59.3	±5.0	57.8	±7.1	0.357	53.6	±10.5	49.8	±11.7	0.227	48.1	±12.8	46.2	±14.4	0.654
Aortic valve area (cm^2^)	0.8	±0.1	0.8	±0.2	0.514	0.8	±0.2	0.8	±0.3	0.268	0.8	±0.2	0.9	±0.1	0.027 *
Mean pressure gradient (mmHg)	54.4	±22.6	58.7	±21.2	0.349	44.4	±14.4	54.2	±24.9	0.081	44.7	±17.5	49.8	±17.2	0.341
PA systolic pressure (mmHg)	34.6	±11.6	38.6	±14.1	0.417	43.4	±14.8	39.2	±12.4	0.286	42.4	±15.0	46.2	±11.8	0.365
Mitral regurgitation					0.482					0.017 *					0.124
0	1	(5.0%)	2	(5.4%)		0	(0%)	1	(3.6%)		1	(2.2%)	0	(0%)	
1	13	(65.0%)	18	(48.6%)		27	(73.0%)	10	(35.7%)		32	(71.1%)	5	(50.0%)	
2	6	(30.0%)	17	(45.9%)		10	(27.0%)	16	(57.1%)		12	(26.7%)	4	(40.0%)	
3	0	(0%)	0	(0%)		0	(0%)	1	(3.6%)		0	(0%)	1	(10.0%)	
Clinical history															
Diabetes mellitus	4	(20.0%)	15	(40.5%)	0.116	11	(29.7%)	11	(39.3%)	0.420	16	(35.6%)	5	(50.0%)	0.480
Dyslipidemia ^f^	5	(25.0%)	8	(21.6%)	0.754	7	(18.9%)	9	(32.1%)	0.220	13	(28.9%)	2	(20.0%)	0.710
Hypertension	14	(70.0%)	25	(67.6%)	0.850	22	(59.5%)	21	(75.0%)	0.190	35	(77.8%)	7	(70.0%)	0.685
Atrial fibrillation ^f^	4	(20.0%)	6	(16.2%)	0.728	10	(27.0%)	5	(17.9%)	0.385	17	(37.8%)	4	(40.0%)	1.000
Pacemaker	0	(0%)	0	(0%)	---	2	(5.4%)	1	(3.6%)	1.000	1	(2.2%)	0	(0%)	1.000
COPD ^f^	3	(15.0%)	5	(13.5%)	1.000	9	(24.3%)	6	(21.4%)	0.784	12	(26.7%)	4	(40.0%)	0.453
Smoker ^f^	1	(5.0%)	3	(8.1%)	1.000	6	(16.2%)	6	(21.4%)	0.592	6	(13.3%)	2	(20.0%)	0.627
Old Stroke ^f^	2	(10.0%)	2	(5.4%)	0.607	2	(5.4%)	1	(3.6%)	1.000	8	(17.8%)	0	(0%)	0.326
Uremia ^f^	2	(10.0%)	0	(0%)	0.119	0	(0%)	1	(3.6%)	0.431	14	(31.1%)	6	(60.0%)	0.144
Cancer ^f^	3	(15.0%)	0	(0%)	0.039 *	6	(16.2%)	2	(7.1%)	0.449	8	(17.8%)	0	(0%)	0.326
PAOD	0	(0%)	0	(0%)	---	3	(8.1%)	3	(10.7%)	1.000	4	(8.9%)	2	(20.0%)	0.298
Coronary artery disease	7	(35.0%)	9	(24.3%)	0.392	22	(59.5%)	13	(46.4%)	0.297	31	(68.9%)	1	(10.0%)	0.001 **
Previous MI ^f^	2	(10.0%)	1	(2.7%)	0.279	6	(16.2%)	6	(21.4%)	0.592	12	(26.7%)	1	(10.0%)	0.421
s/p CABG	0	(0%)	0	(0%)	---	1	(2.7%)	0	(0%)	1.000	1	(2.2%)	0	(0%)	1.000
Previous PCI ^f^	5	(25.0%)	2	(5.4%)	0.084	19	(51.4%)	7	(25.0%)	0.032 *	26	(57.8%)	1	(10.0%)	0.012 *
PCI in last 12 months ^f^	5	(25.0%)	0	(0%)	0.004 **	13	(35.1%)	4	(14.3%)	0.058	24	(53.3%)	1	(10.0%)	0.015 *
Previous cardiac surgery ^f^	0	(0%)	1	(2.7%)	1.000	0	(0%)	0	(0%)	---	1	(2.2%)	1	(10.0%)	0.333

Mann–Whitney U test. ^f^ = F-test, Chi-Square test. Fisher’s exact test. * *p* < 0.05, ** *p* < 0.01. Continuous data were expressed mean ± SD. Categorical data were expressed as number and percentage. PA = pulmonary artery; COPD = chronic obstructive pulmonary disease; PAOD = peripheral arterial occlusive disease; MI = myocardial infarction; PCI = percutaneous coronary intervention; s/p CABG = status post coronary artery bypass grafting.

**Table 2:** Intraoperative and postoperative outcomes.

	STS Score < 4%					STS Score 4–8%					STS Score > 8%				
Outcomes	TAVI (*n* = 20)		SAVR (*n* = 37)		*p* Value	TAVI (*n* = 37)		SAVR (*n* = 28)		*p* Value	TAVI (*n* = 45)		SAVR (*n* = 10)		*p* Value
Combined CABG	0	(0%)	7	(18.9%)	0.045 *	0	(0%)	6	(21.4%)	0.005 **	0	(0%)	0	(0%)	---
Intraoperative Complications ^f^															
Paravalvular leak	8	(40.0%)	0	(0%)	<0.001 **	10	(27.0%)	0	(0%)	0.007 **	19	(42.2%)	0	(0%)	0.010 *
Coronary occlusion	0	(0%)	0	(0%)	---	1	(2.7%)	0	(0%)		0	(0%)	0	(0%)	---
Major bleeding	0	(0%)	0	(0%)	---	2	(5.4%)	3	(10.7%)	0.644	8	(17.8%)	0	(0%)	0.326
Stoke	0	(0%)	0	(0%)	---	0	(0%)	0	(0%)	---	1	(2.2%)	1	(10.0%)	0.333
Major vascular complication ^f^	1	(5.0%)	0	(0%)	0.351	3	(8.1%)	1	(3.6%)	0.628	3	(6.7%)	0	(0%)	1.000
Postoperative Complications															
New pacemaker ^f^	3	(15.0%)	2	(5.4%)	0.332	6	(16.2%)	2	(7.1%)	0.449	8	(18.2%)	4	(40.0%)	0.203
Re-on Endo ^f^	1	(5.0%)	0	(0%)	0.351	1	(2.7%)	3	(10.7%)	0.307	4	(8.9%)	2	(20.0%)	0.298
Acute kidney injury ^f^	0	(0%)	4	(10.8%)	0.286	7	(18.9%)	7	(25.0%)	0.555	8	(17.8%)	3	(30.0%)	0.400
Extubation in operation room	16	(80.0%)	0	(0%)	<0.001 **	16	(43.2%)	0	(0%)	<0.001 **	12	(26.7%)	0	(0%)	0.096
Ventilator (hour)	4.3	±0.6	31.7	±62.3	0.001 **	36.8	±126.6	474.4	±1678.3	<0.001 **	145.5	±397.8	183.6	±265.1	0.237
ICU stay (h)	107.0	±73.6	76.3	±65.4	0.012 *	356.5	±1432.1	506.6	±1673.9	0.491	265.5	±467.6	268.2	±333.8	0.785
Hospital stay (d)	7.9	±5.9	10.4	±3.8	0.005 **	7.2	±7.7	17.2	±17.2	<0.001 **	15.0	±24.1	22.5	±20.7	0.029*
In-Hospital Mortality ^f^	0	(0%)	1	(2.7%)	1.000	2	(5.4%)	2	(7.1%)	1.000	5	(11.1%)	3	(30.0%)	0.149
Cardiac ultrasonography															
Left ventricular ejection fraction (%)	57.8	±5.5	56.4	±5.1	0.240	53.8	±8.7	55.9	±5.6	0.845	51.9	±9.1	63.0	±2.7	0.002 **
Aortic valve area (cm^2^)	1.8	±0.1	1.7	±0.3	0.265	1.8	±0.3	1.6	±0.3	0.052	1.8	±0.4	1.4	±0.2	0.012 *
Mean pressure gradient (mmHg)	11.6	±4.5	14.8	±7.2	0.191	12.6	±8.6	14.6	±7.5	0.123	12.6	±7.0	15.0	±4.7	0.237
PA systolic pressure (mmHg)	35.1	±9.9	32.6	±7.9	0.478	40.8	±14.8	39.0	±14.4	0.475	44.6	±14.3	42.2	±15.2	0.889
Follow-up time (year)	3.4	±2.3	4.7	±3.2	0.155	2.6	±2.5	4.2	±2.6	0.012 *	1.7	±1.8	2.2	±1.9	0.458
Overall mortality ^f^	2	(10.0%)	8	(21.6%)	0.467	10	(27.0%)	12	(42.9%)	0.182	21	(46.7%)	9	(90.0%)	0.015 *
1-year mortality ^f^	0	(0%)	2	(5.4%)	0.536	4	(10.8%)	2	(7.1%)	0.692	17	(37.8%)	3	(30.0%)	0.731
5-year mortality ^f^	2	(10.0%)	4	(10.8%)	1.000	10	(27.0%)	7	(25.0%)	0.854	21	(46.7%)	7	(70.0%)	0.295
Re-Hospitalization ^f^	2	(10.0%)	8	(21.6%)	0.467	6	(16.2%)	6	(21.4%)	0.592	8	(17.8%)	3	(30.0%)	0.400

Mann–Whitney U test. ^f^ = F-test. Chi-Square test. Fisher’s exact test. * *p* < 0.05, ** *p* < 0.01. Continuous data were expressed mean ± SD. Categorical data were expressed as number and percentage. CABG = coronary artery bypass graft; Endo = endotracheal tube; ICU = intensive care unit.

**Table 3:** Univariate and multivariate risk factors Cox-regression analysis.

	Univariate			Multivariate		
	HR	95%CI	*p* Value	HR	95%CI	*p* Value
In-Hospital Mortality						
Group						
TAVI	1.00					
AVR	0.88	(0.29–2.66)	0.814			
Gender						
Female	1.00			1.00		
Male	1.55	(0.52–4.61)	0.435	1.40	(0.39–5.04)	0.607
STS score (%)						
<4%	1.00			1.00		
4–8%	2.46	(0.27–22.88)	0.428	1.83	(0.18–18.41)	0.610
>8%	3.44	(0.38–30.75)	0.270	2.40	(0.23–25.23)	0.465
LVEF	0.96	(0.93–1.00)	0.062	0.97	(0.93–1.02)	0.283
Diabetes mellitus	3.24	(1.05–9.98)	0.040 *	3.71	(1.12–12.32)	0.032 *
PAOD	2.05	(0.44–9.57)	0.362	2.28	(0.44–11.93)	0.330
1-year Mortality						
Group						
TAVI	1.00					
AVR	0.74	(0.44–1.24)	0.253			
Age	1.07	(1.01–1.13)	0.028 *	1.00	(0.94–1.07)	0.893
Gender						
Female	1.00			1.00		
Male	2.42	(1.14–5.18)	0.022 *	1.88	(0.79–4.49)	0.156
BMI < 20	2.80	(1.23–6.36)	0.014 *	1.03	(0.40–2.67)	0.954
STS score (%)						
<4%	1.00			1.00		
4–8%	2.74	(0.55–13.59)	0.217	1.79	(0.33–9.78)	0.499
>8%	12.57	(2.94–53.83)	0.001 **	6.03	(1.20–30.46)	0.030 *
LVEF	0.97	(0.94–0.995)	0.021 *	0.98	(0.94–1.01)	0.202
Diabetes mellitus	1.25	(0.58–2.66)	0.570			
PAOD	1.94	(0.59–6.44)	0.277			
Re-on Endo	11.20	(4.89–25.66)	<0.001 **	4.19	(1.48–11.89)	0.007 **
Acute kidney injury	5.50	(2.61–11.57)	<0.001 **	4.12	(1.74–9.73)	0.001 **
5-year Mortality						
Group						
TAVI	1.00			1.00		
AVR	0.54	(0.30–0.97)	0.040 *	0.81	(0.38–1.71)	0.576
Age	1.06	(1.01–1.11)	0.015 *	1.01	(0.95–1.08)	0.689
BMI < 20	2.25	(1.18–4.31)	0.014 *	1.52	(0.75–3.09)	0.246
STS score (%)						
<4%	1.00			1.00		
4–8%	2.84	(1.12–7.20)	0.028 *	1.69	(0.61–4.66)	0.311
>8%	8.13	(3.34–19.77)	<0.001 **	3.13	(1.01–9.74)	0.048 *
LVEF	0.97	(0.95–0.99)	0.015 *	0.99	(0.96–1.01)	0.360
Diabetes mellitus	1.54	(0.89–2.68)	0.124			
PAOD	2.91	(1.23–6.88)	0.015 *	2.96	(1.13–7.77)	0.028 *
Re-on Endo	8.30	(3.98–17.29)	<0.001 **	3.87	(1.53–9.82)	0.004 **
Acute kidney injury	3.50	(1.95–6.28)	<0.001 **	3.98	(1.95–8.15)	0.001 **

Cox regression. * *p* < 0.05, ** *p* < 0.01. LVEF = left ventricular ejection fraction; PAOD = peripheral arterial occlusive disease; BMI = body mass index; Endo = endotracheal tube.

## Data Availability

The raw data supporting the conclusion of this article will be made available by the authors, without undue reservation.
